# A De Novo Splicing Mutation of *STXBP1* in Epileptic Encephalopathy Associated with Hypomyelinating Leukodystrophy

**DOI:** 10.3390/ijms252010983

**Published:** 2024-10-12

**Authors:** Zixuan Wang, Jun Zhang, Yunfei Zhou, Guicen Liu, Zixin Tian, Xi Song

**Affiliations:** Department of Cell Biology, and Genetics, Institute of Molecular Medicine, and Oncology, Chongqing Medical University, Chongqing 400016, China; 2022130156@stu.cqmu.edu.cn (Z.W.); 202201578@hrbmu.edu.cn (Y.Z.); guicenliu@stu.cqmu.edu.cn (G.L.); 2019110009@stu.cqmu.edu.cn (Z.T.); songxi@stu.cqmu.edu.cn (X.S.)

**Keywords:** *STXBP1*, early infantile epileptic encephalopathy, whole-exome sequencing, splicing mutation, CRISPR/Cas9, nonsense-mediated mRNA decay, haploinsufficiency, hypomyelinating leukodystrophy

## Abstract

Deleterious variations in *STXBP1* are responsible for early infantile epileptic encephalopathy type 4 (EIEE4, OMIM # 612164) because of its dysfunction in the central nervous system. The clinical spectrum of the neurodevelopmental delays associated with STXBP1 aberrations is collectively defined as *STXBP1* encephalopathy (*STXBP1*-E), the conspicuous features of which are highlighted by early-onset epileptic seizures without structural brain anomalies. A girl was first diagnosed with unexplained disorders of movement and cognition, which later developed into *STXBP1*-E with unexpected leukoaraiosis and late onset of seizures. Genetic screening and molecular tests alongside neurological examinations were employed to investigate the genetic etiology and establish the diagnosis. A heterozygous mutation of c.37+2dupT at the *STXBP1* splice site was identified as the pathogenic cause in the affected girl. The de novo mutation (DNM) did not result in any truncated proteins but immediately triggered mRNA degradation by nonsense-mediated mRNA decay (NMD), which led to the haploinsufficiency of *STXBP1*. The patient showed atypical phenotypes characterized by hypomyelinating leukodystrophy, and late onset of epileptic seizures, which had never previously been delineated in *STXBP1*-E. These findings strongly indicated that the haploinsufficiency of *STXBP1* could also exhibit divergent clinical phenotypes because of the genetic heterogeneity in the subset of encephalopathies.

## 1. Introduction

*STXBP1*, also known as Syntaxin binding protein 1, is a protein that is conserved throughout evolution, and plays a crucial role in the release of neurotransmitters and presynaptic vesicles by interacting with syntaxin, which is a receptor that attaches to the cell membrane [1,2,3]. Mutations in *STXBP1* are typically responsible for a brain disorder known as early infantile epileptic encephalopathy type 4 (EIEE4, OMIM # 612164), which is now altogether referred to as *STXBP1* encephalopathy (*STXBP1*-E). *STXBP1*-E encompasses a wide range of neurological disorders involving the brain [4]. The typical symptoms are characterized by the early onset of epilepsy (i.e., refractory seizures, ongoing epileptiform activity), and moderate to severe intellectual disability in infants within three months of birth. The median age of seizure onset is six weeks, and age ranges from 1 day to 13 years [5]. Seizure types include infantile spasms, generalized tonic–clonic, clonic, or tonic seizures, and myoclonic, focal, atonic absence seizures [5]. Delays in intellectual and physical development are commonly presented along with limb hemiplegia, speech, and cognitive impairment in the affected patients, who thus frequently exhibit varying degrees of intellectual disability and motor difficulties. In previous studies, the electroencephalogram (EEG) revealed a characteristic type of focal epileptic activity, including suppression-burst, hypsarrhythmia, or generalized spike-and-slow waves, while brain magnetic resonance imaging (MRI) examination usually showed no cortical malformation [6,7]. To date, most probands represent simplex cases (i.e., a single occurrence in a family). De novo heterozygous pathogenic variations have accounted for a majority of the disorders [5,8,9].

In this study, a girl was first diagnosed with unexplained delays in both movement and cognition at the age of one year and eight months. However, the girl’s diagnosis subsequently developed into epileptic encephalopathy. Primary genetics screening had been carried out, including the analyses of karyotype, fragile X, Angelman, and Prader–Willi syndrome, all with negative outcomes (Figure S1). In a follow-up (2014), a variation of c.37+2dupT in *STXBP1* had been eventually identified as the putative disease-causing mutation. The de novo mutation was located at the donor splice site, which is adjacent to the first exon. Our findings revealed that the de novo variant caused distinct clinical phenotypes compared to those previously reported in *STXBP1*-E.

## 2. Results

### 2.1. Clinical Characteristics, Course of Disease, and Trio WES

**Figure 1:**
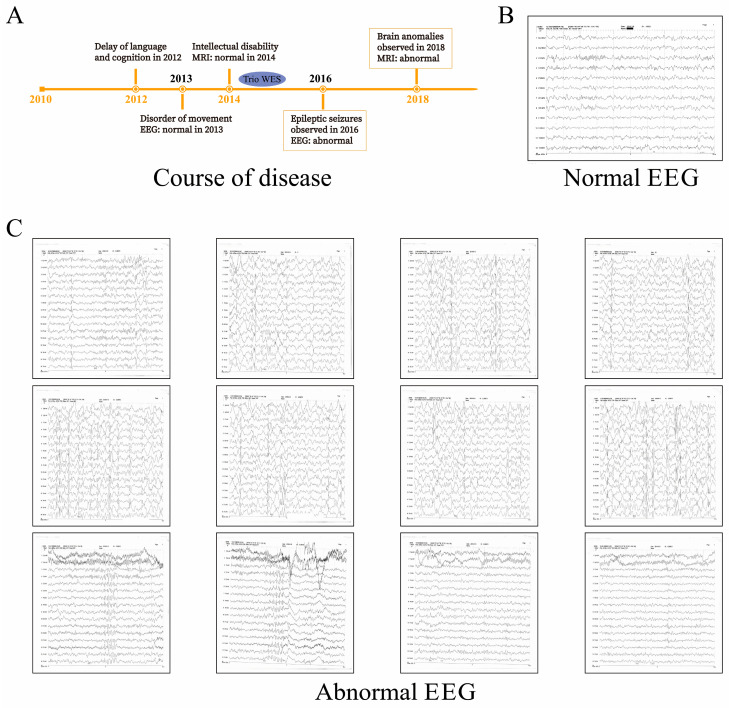
The course of the disease and clinical features. (**A**). Course of the disease. A schematic illustration of the clinical records of the proband from her first visit is presented here. (**B**). Normal EEG. The normal EGG of the girl was recorded at three years old. All * refer to separator. (**C**). Abnormal EEG. Abnormal EGG of the affected girl was first observed at six years old. The EEG was characterized by focal epileptic activity, burst suppression, hypsarrhythmia, or generalized spike-and-slow waves.

**Figure 2:**
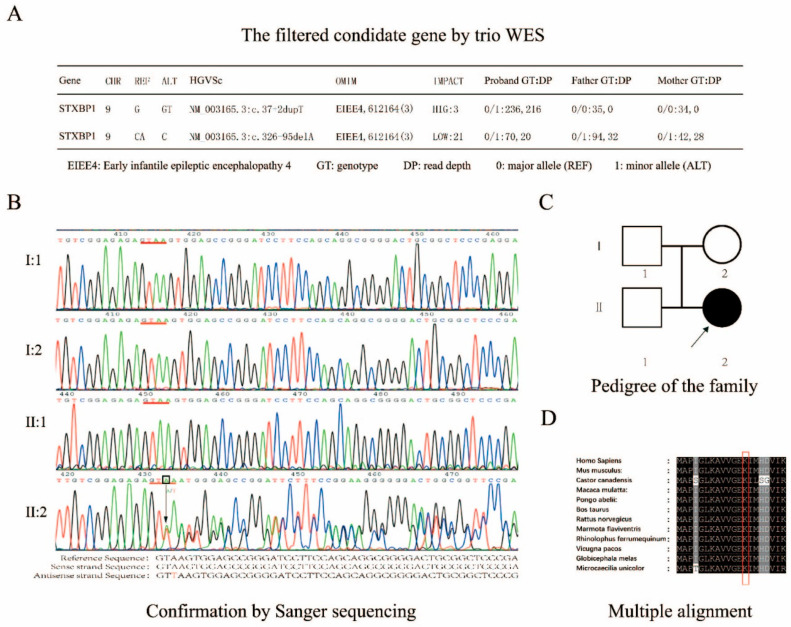
Identification of a pathogenic splice variant of c.37+2T in *STXBP1*. (**A**) The candidate pathogenic variants screened by trio WES. (**B**) Confirmation of the variant by direct Sanger sequencing. (**C**) The pedigree of the family. According to the results, the affected girl harbored the de novo mutation. Arrow marked the proband. (**D**) The multiple alignments of the adjacent amino acid residues to the donor splice site. The adjacent amino acids were highly conserved between species. Red box highlight the amino acid of the mutation site.

The girl first experienced developmental delays at one year and eight months, including growth retardation and movement disorder, as well as cognitive deficiency. Routine neurological examinations had been carried out alongside genetic screening (Figure S1), but the pathological basis was not revealed yet. To investigate pathogenic causality, we conducted a long-term clinical follow-up (Figure 1A). However, the affected girl did not experience epileptic spasms until she was six years old (Figure 1B,C). The observed EEG features showed high similarity to those in typical *STXBP1*-E. Interictal sleep EEG indicated discharges of slow spikes, and waves at 2.5 Hz followed by a hypo-voltage period. The epileptic wave pattern of a distinct burst-inhibition type was the main characteristic presented in the patient’s EEG (Figure 1C). During the period, a de novo heterozygous variation had been identified as the pathogenic mutation associated with *STXBP1*-E (Figure 2A). Although there were two potential pathogenic mutations detected, as a three-person family, the parents and their daughter carried the c.326-95A mutation, but only the daughter showed bad symptoms. The parents are both healthy. This is the most important piece of evidence for excluding the harmfulness of the c.326-95A mutation. The identified variant c.37+2dupT was located at the donor splice site at the junction between exon1 and intron1 of *STXBP1*. None of her healthy siblings and parents or the control participants carried the mutation, which was confirmed by Sanger sequencing (Figure 2B,C). The region comprising the mutation was highly conserved in the sequence between species (Figure 2D).

### 2.2. Bioinformatic Analyses of the Splicing Mutation

The mutation c.37+2dupT was classified as “disease-causing” by in silico analysis with Mutation Taster (Figure S3A). The variant could initiate nonsense-mediated mRNA decay (NMD), which might produce the haploinsufficiency of *STXBP1*. Because the variation occurred at the first exon–intron boundary, exon-skipping did not appear to occur. Based on the three independent splice site predictions above, the presence of c.37+2dupT would affect the donor splicing of *STXBP1* exon1. The nearest downstream donor site from the mutation site was overlapped with the strongest predicted donor site, which was 627 bp downstream of the original cleavage site (Figure S3B,C). However, premature termination codon (PTC, GAGAGTAA→GAGAGTTAA) TAA, caused by insertion of T, would halt the transcription and subsequent processes immediately. The nonsense-mediated mRNA decay (NMD) mechanism was most likely utilized to trigger degradation of the premature mRNA. To further verify whether the mutation induced NMD or not, the novel splicing variation was bioengineered into primary cultured neuron cells by genome editing with CRISPR/Cas9.

### 2.3. Bioengineering in Primary Neuron Cells

**Figure 3:**
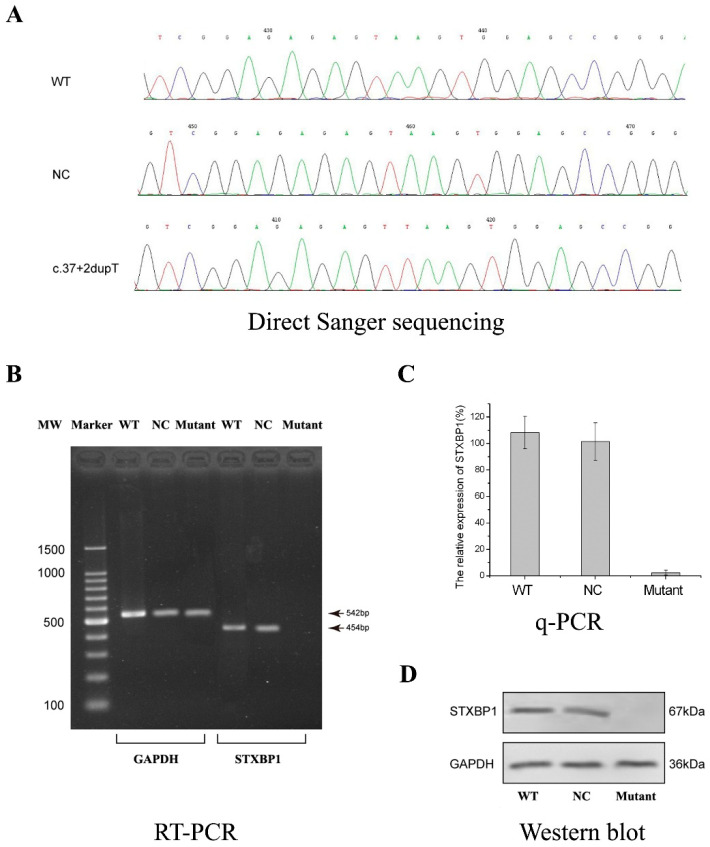
The primary cultured neuron cells were bioengineered by CRISPR/Cas9 technology. (**A**) Confirmation of the bioengineered cell line with c.37+2dupT homogonous mutation in *STXBP1*. (**B**) *STXBP1* mRNA expression detected by RT-PCR. (**C**) The quantification of the *STXBP1* mRNA level by q-PCR. The data were presented as mean ± standard deviation (SD). (**D**) The STXBP1 protein probed by Western blotting.

The primary neuron cells were bioengineered with CRISPR/Cas9 technology, and then generated the desired cell line (Figure 3A). After scrupulous antibiotic screening, we successfully obtained the mutant cell line carrying the homozygous variation of *STXBP1* c.37+2dupT, which was verified by direct Sanger sequencing (Figure 3B). Compared with wild-type neuron cells, the mutant cell line lost the ability to express mature STXBP1 (Figure 3C). Meanwhile, the data indicate the occurrence of the NMD mechanism as mentioned above, and the c.326-95A mutation’s location is too far away from the transcriptional start site. The pathogenicity probability of the c.326-95A mutation is much less than that of the c.37+2dupT mutation. These results are highly consistent with the in silico prediction above, validating that the new mutation could trigger NMD and then invite *STXBP1* haploinsufficiency.

### 2.4. Brain MRI

**Figure 4:**
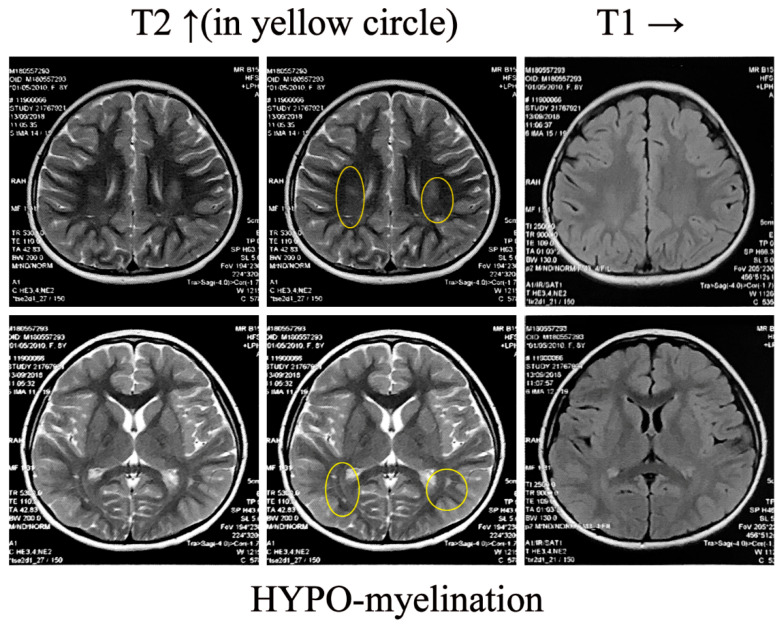
Leukoaraiosis with mild dysmyelination observed in bilateral parietal lobes. Brain MRI from the proband showed diffuse white matter hyperintensity on T2-weighted images (indicated by yellow circles), and the T1-weighted signal represented isointensity, which was consistent with a hypomyelinating leukodystrophy.

Prior to the age of six, the patient exhibited no cortical malformation on her brain MRI, as shown in Figure 1A. Unexpectedly, the girl’s brain MRI began to exhibit mild leukoaraiosis at the age of eight, as depicted in Figure 4. Diffuse white matter with hypomyelination in the parietal lobes was detected in her brain MRI, which lagged behind the late onset of seizures. The MRI confirmed the structural abnormalities in the proband, which rarely occurred in *STXBP1*-E. Suspicious of previous inference, we reanalyzed the trio WES data, especially focusing on the risk genes related to leukodystrophy and leukoencephalopathy, but there were no deleterious variations found. Thus, the clinical manifestation ought to be ascribed to the discerned genetic alteration in *STXBP1*.

## 3. Discussion

STXBP1, also known as Munc-18-1/unc-18/unc18-1, is an essential protein that exhibits a high level of expression in the brain and neuronal tissues. The protein can dock in presynaptic vesicles through its interactions with various SNARE (soluble N-ethylmaleimide-sensitive factor attachment protein receptor) complexes. Among these complexes, syntaxin-1 *(STX-1*) is recognized as the partner subunit. STXBP1 can bind using the open-and-close conformation of syntaxin-1, and cyclically regulate the release of neurotransmitters, and exocytosis [4,10,11]. Despite reductions in protein levels, STXBP1 maintains control over neurotransmitter release rates [12]. Only a complete homozygous deletion of *STXBP1* can lead to synaptic transmission blockade and subsequent neurodegeneration [13,14].

Mutations in the *STXBP1* gene have been associated with severe early infantile epileptic encephalopathies (EIEE), nonsyndromic epilepsy, and moderate to profound cognitive impairment [15,16]. Although patients with *STXBP1*-E share prominent common symptoms, including early onset of seizures, dyskinesia, cognitive impairment, and intellectual disability generally without brain structural abnormality, the clinical spectrum of *STXBP1*-E still expands to include much more extensive neurological disorders [15,16,17]. Related epilepsy syndromes can include but are not limited to Ohtahara syndrome, West syndrome, Lennox–Gaustaut syndrome, Dravet syndrome (not *SCN1A*-related), classic Rett syndrome (not MECP2-related), and atypical Rett syndrome (not *CDKL5*-related). The diagnosis of *STXBP1*-E was always a challenge for clinical neurologists because the clinical phenotypes were very complicated. The mechanism for the phenotypic heterogeneity linked with diverse *STXBP1* mutations is currently unknown and requires further elucidation. It is readily understood that missense mutations occurring in various domains of STXBP1 may have distinct impacts on its functionalities, which in turn present versatile clinical phenotypes due to their dominant-negative effects. However, in previous *STXBP1*-E cases that were caused by splicing mutations or complete deletion of *STXBP1*, the clinical manifestations of haploinsufficiency were highly similar and were highlighted by early onset of seizures without structural brain malformation [17,18,19,20]. While a patient with a gDNA deletion of 2M containing *STXBP1* did exhibit severe cerebral hypomyelination [21], it eventually turned out that the loss of *SPTAN1* in the same region, rather than *STXBP1*, was responsible for the clinical phenotype in the affected subject [19,22].

The girl affected by *STXBP1* haploinsufficiency presented atypical clinical features with late-onset epileptic seizures, and mild leukoaraiosis in addition to the classic symptoms. The severity of *STXBP1*-E usually demonstrated a strong correlation with the type and location of the mutations. In comparison to those carrying dominant-negative variations, the patient exhibited milder clinical symptoms and slower progression. According to previous investigations, most splicing variations could generate an unexpected new premature termination codon (PTC) resulting in a truncated precursor mRNA. Subsequently, the NMD factors were recruited to PTC-containing mRNA, and degraded the abnormal precursor with the help of these factors, forming a “functional complex” [23]. The NMD pathway might have different cleavage activities on PTC-containing mRNA, because the splicing mutations occurred at various loci. Higher degradation activities were probably attributed to a closer distance between the 5′ end of *STXBP1* and the splice variation, which could more effectively suppress the negative effects of the abnormal mRNA compared to on the normal one. According to the data in the ClinVar database, a milder clinical phenotype was observed as the splice variation approached closer to the 5′ end of *STXBP1*. In contrast with the known cases, the affected girl carried the splicing mutation that was closest to the 5′ end of *STXBP1*, presenting much milder clinical phenotypes. Haploinsufficiency caused by the deletion of a gDNA region involving *STXBP1* was different from that caused by splicing mutations at a molecular level, which certainly could not touch off the completely identical clinical symptoms. *STXBP1* haploinsufficiency caused critical damage to not only synaptic transmission in human neurons, but also to neuronal development, dendrite formation, and survival [24]. Although leukoaraiosis had never previously been observed in *STXBP1*-E patients, the paper demonstrated that brain malformation might also be produced by *STXBP1* splicing mutations. The results strongly indicated that *STXBP1* haploinsufficiency with genetic heterogeneity might lead to divergent clinical phenotypes in a subset of encephalopathies.

**Figure 5:**
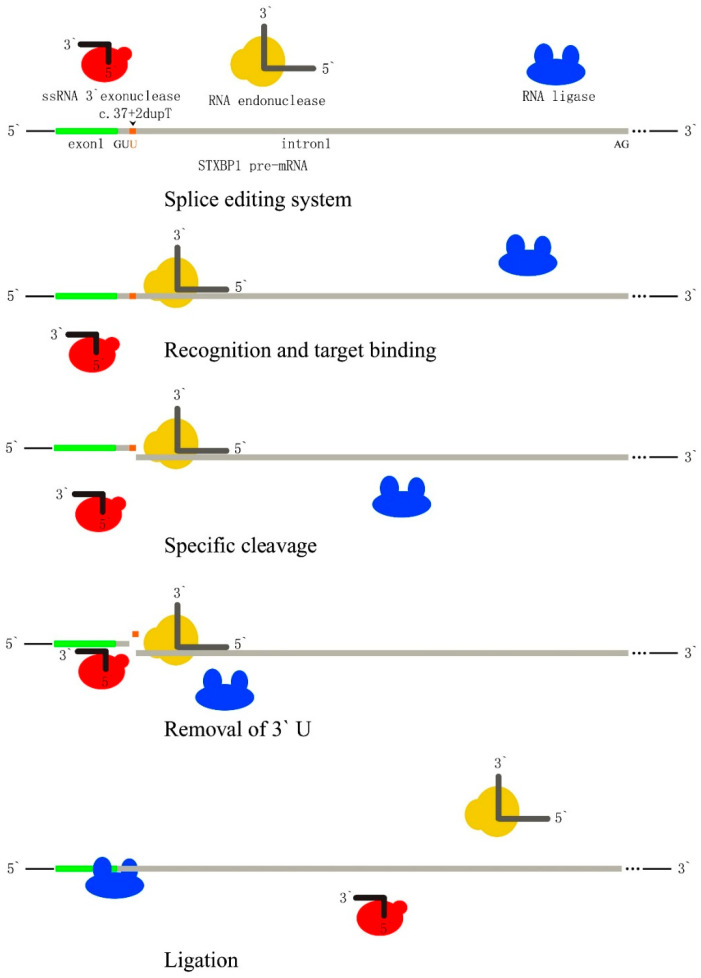
A therapeutic strategy for splice editing involves two key components: a bioengineered RNA endonuclease and exonuclease, both bearing target antisense oligonucleotides. The endonuclease targets the junction between exon 1 and intron 1, breaking the 3,5 phosphate diester bond at the 3′ terminus of the mutant GUU. Then, the RNA exonuclease removes the duplicated Uridine from the target sequence in exon 1. Endogenous RNA editing and repairing enzymes are then able to rejoin the cleaved *STXBP1* pre-mRNA. This approach shows promise for therapeutically correcting splice site mutations. Therapeutic editing in pre-mRNA can share this strategy in correcting different mutations, including missense, deletion, and insertion, with dependence on relevant bioengineered RNA repairing partners. Red bubbles represent ssRNA 3′ exonuclease, yellow bubbles represent RNA endonuclease, blue bubbles represent RNA ligase, green bar represent exon1 of *STXBP1* pre-mRNA and gray bar represent intron1 of *STXBP1* pre-mRNA.

About 11% of all human disease-associated gene lesions are nonsense mutations [25], which frequently occur at the splice junctions in newborns, resulting in the introduction of an in-frame PTC into the protein-coding gene sequence. Therapeutic strategies aimed at correcting the mutations, which might restore the deficient protein function, have the potential to provide a therapeutic benefit for many patients [26]. We suggest a strategy for pre-mRNA splicing–editing through the utilization of the endogenous RNA editing or repairing system in eukaryotes (Figure 5). This interesting regime comprises two key components: a modified RNA endonuclease and exonuclease, both capable of recognizing specific sequences and binding. Most of the mRNAs carrying a PTC can be rapidly degraded by the surveillance mechanism of NMD, thus decreasing the levels of PTC-containing mRNAs in the cell, and their availability for splice mutation correcting. Accordingly, the use of NMD inhibitors might greatly augment the efficiency of therapeutic splice editing. This approach could be effective in achieving the desired outcomes but requires further research and testing. At present, gene-editing technology is not only used as a scientific research tool, but also as a clinical observation and treatment method to solve practical problems for patients. The ethical issues that exist in the process of designing applications can indeed become obstacles. However, the maturity of gene-editing animal technology will greatly solve this problem. Thousands of gene products have undergone repeated confirmation experiments in animal models, benefiting clinical patients, and there are countless examples of this. In terms of our idea, there have been many successful cases at the molecular, cellular, and animal levels of guiding and intervening in the mRNA splicing process, such as induced pluripotent stem cell differentiation technology and gene vaccine immunization of mice. We are confident that we can continuously elucidate this mechanism within a standardized ethical framework, confirm our assumption, and overcome this clinical challenge as soon as possible.

## 4. Materials and Methods

### 4.1. Patient and Participants

The proband was diagnosed with unexplained delay of movement and cognition on her first visit in 2012 at one year and eight months old. The girl was not born from a consanguineous marriage. According to the clinical investigation, both the pregnancy and delivery were uneventful. Head circumference, weight, and length were normal at birth. The girl later developed encephalopathy with epilepsy. In a follow-up, we collected blood samples from all the family members, and 1521 healthy Chinese individuals from Medical Examination Center after obtaining informed consent. The healthy individuals selected as controls were recruited in a random manner, and all participants were at least 18 years of age. The clinical specimens were obtained in accordance with the Declaration of Helsinki and the ethics guidelines of the World Health Organization (WHO).

### 4.2. Whole-Exome Sequencing and Bioinformatics Analyses

Genomic DNA (gDNA) was extracted from peripheral blood leukocytes using a blood DNA extraction kit according to the manufacturer′s instructions (TianGen, Beijing, China). Trio Whole-Exome Sequencing (WES) was conducted on leukocyte gDNAs that were extracted from the peripheral blood, and then they underwent target enrichment with SureSelect Human All Exon V6 Kit (Agilent, Shenzhen, China). Protocols and more details (base calling, variant filtering, bioinformatic analyses, etc.) are shown in the Supplementary Materials (Figure S2). The software packages used in harmfulness prediction to infer the potential functional changes from the variant sequence were strictly in accordance with ACMG (American College of Medical Genetics, and Genomics) standards [27]. Filtered variations (Indel or SNV) linked with the clinical phenotypes were confirmed by PCR–Sanger sequencing. Leukocyte gDNAs were used as the templates for PCR amplification of the target region with the forward primer 5′-CAGCACCGACGGGAAAGA-3′, and the reverse primer 5′-TGAAGGGAAGAGAGGGAGGG-3′. The amplified products were recovered from the agarose gel, and then purified to send out to the Shenzhen HuaDa Gene Research Institute for Sanger sequencing. To analyze the donor-splicing alterations, three in silico tools were used, including Berkeley Drosophila Genome Project (BDGP), NETGene2, and Softberry-FSPLICE. The web resources of the bioinformatic tools are listed in Table S1.

### 4.3. Primary Culture of Neuron Cells

Human primary cortical neuron cells were isolated from fetal brain tissue from a spontaneous abortion at 20 weeks of embryonic age after informed consent was obtained. Following the removal of meninges tissue, the brain tissue was rinsed three times with Hank’s Balanced Salt Solution (HBSS), and then digested with 0.025% trypsin in HBSS for 10–20 min at 37 °C. The digestion was terminated by the direct addition of 20 mL complete medium after the trysinized solution was transferred into a 50 mL test tube. The suspension was passed through a siliconized Pasteurized pipette to mash the tissue for obtaining a single-cell suspension. After centrifuging the cell suspension and discarding the supernatant, the sediment was resuspended in the medium, and seeded under standard culture conditions at a density of 2.5–3.0 × 10^6^ cells/mL on 3.5 cm dishes that were coated with poly-L-lysine. Cytarabine (Ara-C) was added into the dishes at a final concentration of 5 μM after 24 h to inhibit glial cell proliferation, whereas the medium was removed and half-exchanged every 48 h. The cells were cultured in a saturated humidified atmosphere containing 5% CO_2_ at 37 °C in DMEM/F12 medium supplemented with a cocktail formula, including 15% fetal bovine serum, 5 mM glutamine, 10 mM HEPES, 2.2 g/L NaHCO_3_, 30 mM glucose, 100 mU/L insulin, 1 μg/mL antifungal fungizone, and an antibiotics mixture that comprised 10 μg/mL gentamycin, 100 units/mL penicillin, and 100 μg/mL streptomycin.

### 4.4. Genome Editing with CRISPR/Cas9

The CRISPR/Cas9 technology was utilized to perform genome bioengineering on human primary cultured neuron cells. dsDNA (double-stranded DNA) with sticky ends, which encoded the intended target sequences of sgRNA (single-guided RNA), were chemically synthesized, and then cloned into the pSpCas9 (BB)-2A-Puro (PX459) V2.0 plasmid after being digested by BbsI (NEB), conforming to the instructions. The cultured neuron cells were plated into 6 cm poly-L-lysine coated dishes at 70% confluency, and then co-transfected with 5 μg pSpCas9 plasmid carrying sgRNA codes and equal molar ssODN (single-stranded oligodeoxynucleotide) as donor DNA harboring the mutation site. According to the manufacturer’s recommended protocol, co-transfection was performed using a 2:1 ratio of Lipofectamine3000 (Life Technologies, Carlsbad, CA, USA) to vector DNA and ssODN. Puromycin selection started 24 h after transfection. The individualized cell colonies were carefully picked after antibiotic screening for 5–7 days, and then continuously cultured in the coated 96-well plates. The monocolony-derived cells were collected once they were growing to 70% confluency in each well, half of which were used for gDNA isolation. The target region was amplified using the gDNA as a template under the standard PCR reaction conditions. The amplified products were recovered from the gels, and then identified by Sanger sequencing. The sequences of sgRNA-encoding dsDNA, ssODN, and specific primers are displayed in the Supplementary Materials (Table S2).

### 4.5. The Expression of STXBP1 mRNA

Total RNA was extracted from the primary neuron cells using RNeasy Mini Kit (Qiagen, Valencia, CA, USA), and 2 μg RNA was converted into cDNA using SuperScript III First Strand Synthesis Super Mix kit (Life Technologies). The primers for subsequent PCR amplification were designed as follows: RT-PCR for *GAPDH* (F: 5′-GAGCCAAAAGGGTCATCATCTC-3′, R: 5′-AAAGGTGGAGGAGTGGGTGTC-3′) *STXBP1* (F: 5′-CCCGGTCTCTGAAAGATTTTTCTTC-3′, R: 5′-GTGGAATCGGTGACGATGGG-3′); and qRT-PCR for *GAPDH* (F: 5′-GGGAAACTGTGGCGTGAT-3′, R: 5′-GAGTGGGTGTCGCTGTTGA-3′) *STXBP1* (F: 5′-ACTCTGCCGAGTGGAGCA-3′, R: 5′-GTGGAATCGGTGACGATGGG-3′). SYBR Green Supermix with ROX (BioRad, Hercules, CA, USA) was used to perform quantitative RT-PCR. The PCR amplification conditions were set as follows: pre-denaturation at 94 °C for 3 min followed by denaturation at 94 °C for 30 s, annealing at 60 °C for 30 s, elongation at 72 °C for 30 s repeated in 34 cycles, and at 72 °C for an additional 10 min to repair the termini of the fragments upon thermal cycle cessation.

### 4.6. Western Blot

The cultured neuron cells were lysed in RIPA buffer in the presence of a cocktail of protease inhibitors comprising PMSF (100:1) (Beyotime, Shanghai, China, PI021) for 30 min, and then centrifuged for another 30 min. Supernatant was mixed with SDS-PAGE sample loading buffer (Beyotime, P0015) at a ratio of 4:1 before being boiled for 10 min. Protein concentrations were measured using the BCA protein assay kit (Beyotime, P0010). Equivalent amounts of total lysate were separated by 10% sodium dodecyl sulfate polyacrylamide gel electrophoresis (SDS-PAGE), and then transferred from the gel to 0.45 μm polyvinylidene fluoride (PVDF) membrane (Beyotime, FFP39). Subsequently, the membranes were blocked with 5% BSA, and then incubated with the anti-*STXBP1* monoclonal antibody (Abcam, Cambridge, UK, ab124920) and anti-GAPDH monoclonal antibody (Abcam, ab181602) overnight at 4 °C, respectively. Following the incubation, the membranes were quickly washed twice with ice TBST, and then incubated with peroxidase-conjugated secondary antibody (Abcam, ab97051). The blots were screened by ECL (Thermo Scientific, Waltham, MA, USA, 34094) and analyzed with an image acquisition system.

### 4.7. Brain Magnetic Resonance Imaging (MRI) and Electroencephalography (EEG)

Brain MRIs were performed to evaluate structural cortical abnormalities. The EEGs were recorded for analyzing the epileptiform activity and background rhythm disorder. Both examinations were conducted in strict compliance with the routine clinical procedures in a double-blind manner.

## 5. Conclusions

The findings strongly indicated that the haploinsufficiency of *STXBP1* could also exhibit divergent clinical phenotypes because of the genetic heterogeneity in the subset of encephalopathies. Although haploinsufficiency is a universal etiology due to de novo heterozygous mutation, the clinical phenotypes can exhibit multiple facets. We suggest that more attention should be paid to the diversity of clinical phenotypes caused by the haploinsufficiency of disease-related genes in different genetic contexts.

## Data Availability

The datasets generated during and/or analyzed during the current study are available from the corresponding author on reasonable request.

## Supplementary Materials

The following supporting information can be downloaded at https://www.mdpi.com/article/10.3390/ijms252010983/s1.
