# Supplementary material for: A De Novo Splicing Mutation of STXBP1 in Epileptic Encephalopathy Associated with Hypomyelinating Leukodystrophy
Source: Int J Mol Sci. 2024 Oct 12;25(20):10983. doi: 10.3390/ijms252010983 (PMC11507417; doi:10.3390/ijms252010983)
Supplement: Supplementary file 1 [file ijms-25-10983-s001.zip › Submitter - ClinVar.pdf]

Institute of Molecular Medicine and Oncology (Chongqing Medical University)

General information

Institute of Molecular Medicine and Oncology
Chongqing Medical University
Medical School Road 1#, Yuzhong District
Chongqing
China - 400016
https://www.cqmu.edu.cn/
Organization ID: 507731

Personnel

Jun Zhang, Lab Director
Phone: 0086-023-68485806
Email: zhangjun1017cq@sohu.com

Assertion criteria

Level: Assertion criteria provided

ACMG Guidelines, 2015

Summary of submissions to ClinVar

Total submissions: 1

Gene

Gene	Submissions	Last Updated
STXBP1	1	Sep 15, 2020

Condition

Name	Submissions	Last Updated
Developmental and epileptic encephalopathy, 4	1	Sep 15, 2020

Feedback
