# Supplementary material for: A De Novo Splicing Mutation of STXBP1 in Epileptic Encephalopathy Associated with Hypomyelinating Leukodystrophy
Source: Int J Mol Sci. 2024 Oct 12;25(20):10983. doi: 10.3390/ijms252010983 (PMC11507417; doi:10.3390/ijms252010983)
Supplement: Supplementary file 1 [file ijms-25-10983-s001.zip › Supplementary figure captions.pdf]

Captions

Figure S1 Routine genetic testing. A G-banded karyotype analysis. B. The genome-wide copy number variation screening. C. Chromosomal microarray analysis (Affymetrix Cytoscan HD 750K/Optima). D. The molecular diagnosis for fragile X syndrome (FXS) in both FMR1 and FMR2. The CGG repeats number was 29/30 without the pathogenic variation of I304N in FMR1 and that of GCC repeats was 23/23 in FMR2. These results indicated that the affected girl did not carry FXS involved variations.

Figure S2 The schematic illustration for the standard trio Whole Exome Sequencing (WES). A. The flowchart of the WES procedure. B. The workflow of the WES data processing. C. The insert size distribution of the paired reads. D. The statistic summary of the highthrough-put data and quality control.

Figure S3 The deleterious prediction of the splice donor variation. A. Harmfulness prediction by Mutation taster. B. The splice site prediction of both the wild type and mutant sequences with three distinct *in silico* software. C. The red border indicates the position of donor sites. The red arrow refers to the stop codon. I: normal spliced product; II: intron retention; III: nearest alternative spliced variant; IV: another spliced variant. The predicted results were consistent with each other. The splice variation might result in a premature termination due to the creation of a stop codon downstream of the exon 1.
