# Supplementary material for: A De Novo Splicing Mutation of STXBP1 in Epileptic Encephalopathy Associated with Hypomyelinating Leukodystrophy
Source: Int J Mol Sci. 2024 Oct 12;25(20):10983. doi: 10.3390/ijms252010983 (PMC11507417; doi:10.3390/ijms252010983)
Supplement: Supplementary file 1 [file ijms-25-10983-s001.zip › Supplementary Materials and Methods.pdf]

Materials and Methods

Patient and participants

The proband was diagnosed with unexplained delay of movement and cognitive on her first visit in 2012 at one-year-eight-month old. The girl was not born from a consanguineous marriage. Both the pregnancy and delivery were not uneventful. Head circumference, weight and length were normal at birth. The girl later progressed into encephalopathy with epilepsy. In follow-up, we collected blood samples from all the family members and 1521 healthy Chinese individuals from Medical Examination Center after informed consent. These healthy controls were recruited randomly and all were over 18 years old. The clinical specimens were obtained in well accordance with the Declaration of Helsinki and the ethics guidelines of the World Health Organization (WHO).

Whole-Exome Sequencing and bioinformatics analyses

Genomic DNA (gDNA) was extracted from peripheral blood leukocytes using a blood DNA extraction kit according to the manufacturer's instructions (TianGen，Beijing，China). Trio Whole-Exome Sequencing（WES）was performed on the leukocytes DNA of the proband and her parents by target enrichment with SureSelect Human All Exon V6 Kit (Agilent, Shenzhen, China). Protocols and more details (base calling, variant filtering, bioinformatic analyses and etc.) are shown in the supplementary materials (Fig. S2). The software packages used for harmfulness prediction to infer the potential functional changes from the variant sequence were strictly abided by ACMG (American College of Medical Genetics and Genomics) standards [9]. Filtered variations (Indel or SNV) linked with the clinical phenotypes were confirmed by PCR-Sanger sequencing. gDNAs were used as the templates for the PCR amplification of the target region with forward primer: 5’-CAGCACCGACGGAAAGA-3’ and the reverse primer: 5’-TGAAGGGAAGAGAGGGAGGG-3’. The amplified products were recovered from the agarose gel and purified to send out directly to the Shenzhen HuaDa Gene Research Institute for Sanger sequencing. To analyze the donor-splicing alternations, three in silico tools were used, including Berkeley Drosophila Genome Project (BDGP), NETGene2, Softberry-FSPLICE. The web resources of the bioinformatic tools were listed in Table S1.

Primary culture of neuron cells

Human primary cortical neuron cells were isolated from spontaneous abortion fetal brain tissue at the embryonic age of 20 weeks after informed consent. When removal of the tissue meninges followed by thrice rinses with Hank's Balanced Salt Solution (HBSS), the brain tissue was digested with 0.025% HBSS-trypsin at 37 °C for 10-20 minutes. The digestion was terminated by direct addition of 20 ml complete medium after the trysinized solution was transferred into a 50 ml test tube. The suspension was passed through a siliconized Pasteurized pipette to mash the tissue for obtaining the single-cell suspension. After the cell suspension was centrifuged with the supernatant

discarded, the cell sediment was resuspended in the medium and plated on 3.5cm poly-L-lysine coated dishes at the density of 2.5-3.0×10^6/ml under the standard culture conditions. Cytosine arabinoside (Ara-C, final concentration 5μM) were added into the dishes after 24 hours to reduce glial proliferation. The medium was removed and half-exchanged every two days. The cultured cells were maintained in DMEM/F12 medium supplemented with 15% fetal bovine serum, 5mM glutamine, 10mM HEPES, 2.2g/L $NaHCO_3$, 30mM glucose, 100mU/L insulin, 1 μg/ml antifungal fungizone and antibiotics cocktail including 10 μg/ml gentamycin, 100 units/ml penicillin, and 100 μg/ml streptomycin in a saturated humidified atmosphere containing 5% CO_2 at 37 °C.

Genome editing with CRISPR/Cas9

The genome bioengineering of human primary cultured neuron cells was performed using CRISPR/Cas9 technology. The dsDNA (double-stranded DNA) with sticky ends were chemically synthesized in relevance to the intended target sequences of sgRNA (single-guided RNA) and then cloned into the pSpCas9 (BB)-2A-Puro (PX459) V2.0 plasmid after digested by BbsI (NEB) conforming to the instruction. The culture neuron cells were plated into 6 cm poly-L-lysine coated dishes at 70% confluency and co-transfected with 5μg pSpCas9 plasmid carrying sgRNA codes and equal molar ssODN (single-stranded oligodeoxynucleotide) as donor DNA harboring the mutation site using Lipofectamine 3000 (Life Technologies). According to the manufacturer's recommended protocol, transfection was performed using a 2:1 ratio of Lipofectamine to vector DNA and ssODN. Puromycin selection started 24 hours after transfection. The individualized cell colonies were carefully picked after antibiotic screening for 5-7 days and then continuously cultured in the coated 96-well plates. Half of the monoclonal derived cells in each well were collected for gDNA isolation and PCR amplification on the target region until grew to 70% confluency. Subsequently, the PCR products were recovered from the gels and identified by Sanger sequencing. The sequences of sgRNA-encoding dsDNA, ssODN and specific primers were displayed in the supplementary materials (Tab. S2).

The expression of STXBP1 mRNA

Total RNA was extracted from the primary neuron cells using RNeasy Mini Kit (Qiagen, Valencia, CA). 20 μg RNA was converted into cDNA using SuperScript III First Strand Synthesis Super Mix kit (Life Technologies). The primers for subsequent PCR amplification were designed as follows: RT-PCR for GAPDH (F: 5'-GAGCCAAAAGGGTCATCATCTC-3', R: 5'-AAAGGTGGAGGAGTGGGTGTC-3') and STXBP1 (F: 5'-CCCGGTCTCTGAAAGATTTTTCTTC-3', R: 5'-GTGGAATCGGTGACGATGGG-3'); qRT-PCR for GAPDH (F: 5'-GGGAAACTGTGGCGTGAT-3', R: 5'-GAGTGGGTGTCGCTGTTGA -3') and STXBP1 (F: 5'-ACTCTGCCGAGTGGAGCA-3', R: 5'-GTGGAATCGGTGACGATGGG-3'). SYBR Green Supermix with ROX (BioRad) was used to perform quantitative RT-PCR. The PCR amplification conditions were set

as follows: pre-denaturation at 94°C for 3 min followed by denaturation at 94°C for 30 s, annealing at 60°C for 30 s, elongation at 72°C for 30 s with repeated 34 cycles and 72°C for an additional 10 min to repair the termini of the fragments upon the thermal cycle cessation.

Western blot

The cultured neuron cells were lysed in RIPA buffer in the presence of a cocktail of protease inhibitors comprising PMSF (100:1) (Beyotime, PI021) for 30 minutes and sequentially centrifuged for another 30 minutes. Supernatant was mixed with SDS-PAGE sample loading buffer (Beyotime, P0015) at a ratio of 4:1 before boiled for 10 minutes. Protein concentrations were measured using BCA protein assay kit (Beyotime, P0010). Equal amounts of total lysate were analyzed by 10% sodium dodecyl sulfate polyacrylamide gel electrophoresis (SDS-PAGE). Proteins samples were transferred from the gel to 0.45μm polyvinylidene fluoride (PVDF) membrane (Beyotime, FFP39). Membranes were blocked with 5% BSA and incubated with the anti-STXBP1 monoclonal antibody (Abcam, ab124920) and anti-GAPDH monoclonal antibody (Abcam, ab181602) overnight at 4 °C respectively. Following the incubation, the membranes were quickly washed twice with ice TBST and then incubated with peroxidase-conjugated secondary antibody (Abcam, ab97051). The blots were screened by ECL (Thermo Scientific, 34094) and analyzed with an image acquisition system.

Brain magnetic resonance imaging (MRI) and electroencephalography (EEG)

Brain MRIs were performed to evaluate the structural cortical abnormalities. The EEGs were recorded to analyzing the epileptiform activity and background rhythm disorder. Both examinations were conducted in strict compliance with routine clinical procedures in a double blind manner.
