# Supplementary material for: A De Novo Splicing Mutation of STXBP1 in Epileptic Encephalopathy Associated with Hypomyelinating Leukodystrophy
Source: Int J Mol Sci. 2024 Oct 12;25(20):10983. doi: 10.3390/ijms252010983 (PMC11507417; doi:10.3390/ijms252010983)
Supplement: Supplementary file 1 [file ijms-25-10983-s001.zip › Table S1.pdf]

Table S1 Bioinformatic tools

Software	source
**BWA**	https://sourceforge.net/projects/bio-bwa/files/
**Picard（v2.5.0）**	http://broadinstitute.github.io/picard
**GATK(4.1.4.1)**	https://software.broadinstitute.org/gatk/
**SnpEff**	http://snpeff.sourceforge.net/
**SIFT**	https://sift.bii.a-star.edu/sg/
**MutationTaster**	http://www.mutationtaster.org/
**PolyPhen2**	http:genetics.bwh.harvard.edu/pph2/
**Condel**	https://omictools.com/condel-tool
**FATHMM**	http://fathmm.biocompute.org.uk/
**BDGP**	http://www.fruitfly.org/seq_tools/splice.html
**Softberry-FSPLICE**	http://www.softberry.com/berry.phtml?topic=fsplice&group=programs&subgroup=gfind
**NETGENE2**	https://services.healthtech.dtu.dk/service.php?NetGene2-2.42
