# Supplementary material for: A De Novo Splicing Mutation of STXBP1 in Epileptic Encephalopathy Associated with Hypomyelinating Leukodystrophy
Source: Int J Mol Sci. 2024 Oct 12;25(20):10983. doi: 10.3390/ijms252010983 (PMC11507417; doi:10.3390/ijms252010983)
Supplement: Supplementary file 1 [file ijms-25-10983-s001.zip › Table S2.pdf]

Table S2. The corresponding sequences used in CRISPR/Cas9 editing

Designed nucleotides	Sequence
sgRNA1:	5`-CACCGCGCGCGGCGCGTGAGGTG-3`<br>3`–CGCGCGCCGCGCACTCCACCAAA–5`
sgRNA2:	5`–CACCGCGCGCGGCGCGTGAGGTGA-3`<br>3`–CGCGCGCCGCGCACTCCACTCAAA–5`
ssODN/donor DNA:	GGTACCCTGCGCCCCGGGCGCGCGCGGCGCGTGA<br>GGTTGAGGGGGAGGGCGCGGCTCCGCACCAGCC<br>AGCGGC
The PCR primers:	F: 5`–CAGCACCGACGGAAAGA-3`<br>R: 5`- TGAAGGGAAGAGAGGGAGGG-3`
