# Supplementary material for: A De Novo Splicing Mutation of STXBP1 in Epileptic Encephalopathy Associated with Hypomyelinating Leukodystrophy
Source: Int J Mol Sci. 2024 Oct 12;25(20):10983. doi: 10.3390/ijms252010983 (PMC11507417; doi:10.3390/ijms252010983)
Supplement: Supplementary file 1 [file ijms-25-10983-s001.zip › VCV000870412.2 - ClinVar - NCBI.pdf]

ClinVar Genomic variation as it relates to human health

About Access Submit Stats FTP Help new search queries using location, c-dot, and p-dot helpful?

NM_001032221.6(STXBP1):c.37+2dup

Cite this record

Interpretation: **Pathogenic/Likely pathogenic**

Review status: ★★☆☆ criteria provided, multiple submitters, no conflicts

Submissions: 2

First in ClinVar: May 4, 2020

Most recent Submission: Sep 27, 2020

Last evaluated: Aug 6, 2020

Accession: VCV000870412.2

Variation ID: 870412

Description: 1bp duplication

Feedback

Variant details

NM_001032221.6(STXBP1):c.37+2dup

Allele ID: 858591

Variant type:

Duplication

Variant length:

1 bp

Cytogenetic location:

9q34.11

Genomic location:

9: 127612441-127612442 (GRCh38) GRCh38 UCSC
9: 130374720-130374721 (GRCh37) GRCh37 UCSC

HGVS:

Nucleotide	Protein	Molecular consequence
NM_001032221.6:c.37+2dup MANE SELECT		splice donor
NM_001374306.2:c.37+2dup		splice donor
NM_001374307.2:c.-101+2dup		splice donor

... more HGVS

Protein change:

-

Other names:

-

Canonical SPDI:

NC_000009.12:127612441:T:TT

Functional consequence:

sequence variant affecting splice donor [Sequence Ontology SO:1000072]

Global minor allele frequency (GMAF):

-

Allele frequency:

-

Links:

dbSNP: rs1838431452
VarSome

Feedback

Conditions

Aggregate interpretations per condition

Interpreted condition	Interpretation	Number of submissions	Review status	Last evaluated	Variation/condition record
Developmental and epileptic encephalopathy, 4	Pathogenic/Likely pathogenic	2	criteria provided, multiple submitters, no conflicts	Aug 6, 2020	RCV001089985.2

Clinical features observed in individuals with this variant

Epileptic encephalopathy
Global developmental delay
Dystonia

Gene(s)

Gene	OMIM	ClinGen Gene Dosage Sensitivity Curation		Variation viewer	Related variants	
		HI score	TS score		Within gene	All
STXBP1		Sufficient evidence for dosage pathogenicity	No evidence available	GRCh38<br>GRCh37	783	840

Submitted interpretations and evidence

Interpretation (Last evaluated)	Review status (Assertion criteria)	Condition (Inheritance)	Submitter	More information
Likely pathogenic (Jun 09, 2018)	criteria provided, single submitter (ACMG Guidelines, 2015) Method: clinical testing	Developmental and epileptic encephalopathy, 4<br>Affected status: yes<br>Allele origin: de novo	Victorian Clinical Genetics Services,Murdoch Childrens Research Institute<br>Accession: SCV001244977.1<br>First in ClinVar: May 04, 2020<br>Last updated: May 04, 2020	Comment: A heterozygous duplication variant, NM_003165.3(STXBP1):c.37+2dupT, has been identified in intron 1 of 19 of the STXBP1 gene. This variant alters the conserved splice donor recognition … (more)
Pathogenic (Aug 06, 2020)	criteria provided, single submitter (ACMG Guidelines, 2015) Method: clinical testing	Developmental and epileptic encephalopathy, 4 (Autosomal dominant inheritance)<br>Affected status: yes<br>Allele origin: de novo	Institute of Molecular Medicine and Oncology,Chongqing Medical University<br>Accession: SCV001431535.1<br>First in ClinVar: Sep 27, 2020<br>Last updated: Sep 27, 2020	Comment: We observed a young female patient with a de novo variant NM_003165.6 c.37+2dup of STXBP1.Her clinical symptoms overlapped with symptoms of STXBP1-E, including seizures, intellectual … (more)

Feedback

Functional evidence

Functional consequence	Method	Result	Submitter	Supporting information (See all)

Functional consequence	Method	Result	Submitter	Supporting information (See all)
sequence variant affecting splice donor (SO:1000072)			Institute of Molecular Medicine and Oncology,Chongqing Medical University<br>Accession: SCV001431535.1<br>Submitted: (Sep 15, 2020)	Evidence details

Citations for this variant

There are no citations in ClinVar for this variation. If you know of citations for this variation, please consider submitting that information to ClinVar.

Text-mined citations for rs1838431452 none

These citations are identified by LitVar using the rs number, so they may include citations for more than one variant at this location. Please review the LitVar results carefully for your variant of interest.

Record last updated Apr 24, 2022

Feedback

Connect with NLM

National Library of Medicine
8600 Rockville Pike
Bethesda, MD 20894

Web Policies
FOIA
HHS Vulnerability Disclosure

Help
Accessibility
Careers
