# Supplementary figures and images for: A De Novo Splicing Mutation of STXBP1 in Epileptic Encephalopathy Associated with Hypomyelinating Leukodystrophy
Source: Int J Mol Sci. 2024 Oct 12;25(20):10983. doi: 10.3390/ijms252010983 (PMC11507417; doi:10.3390/ijms252010983)

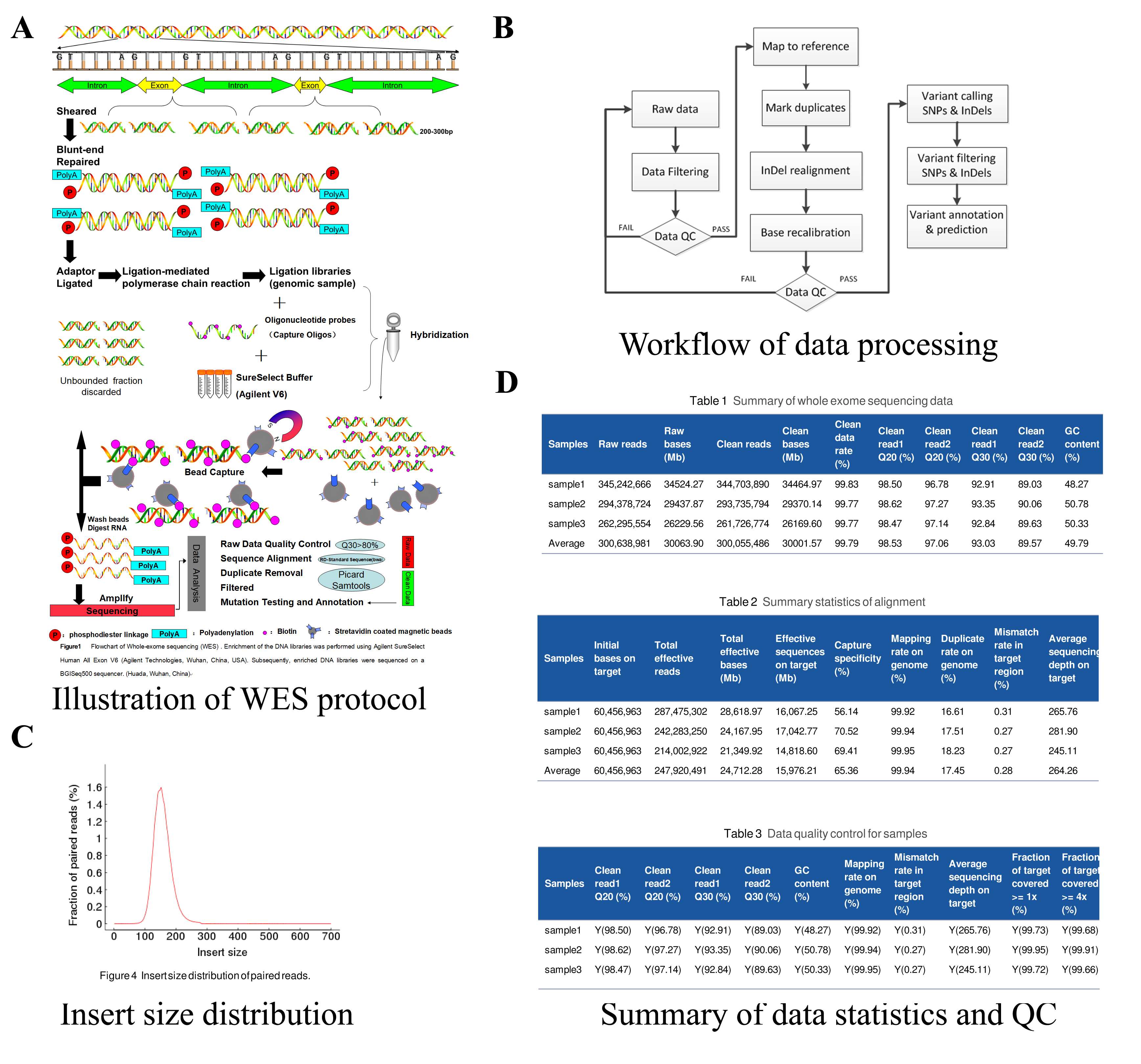

Supplement: Supplementary file 1 [file ijms-25-10983-s001.zip › Figure S2.jpg]

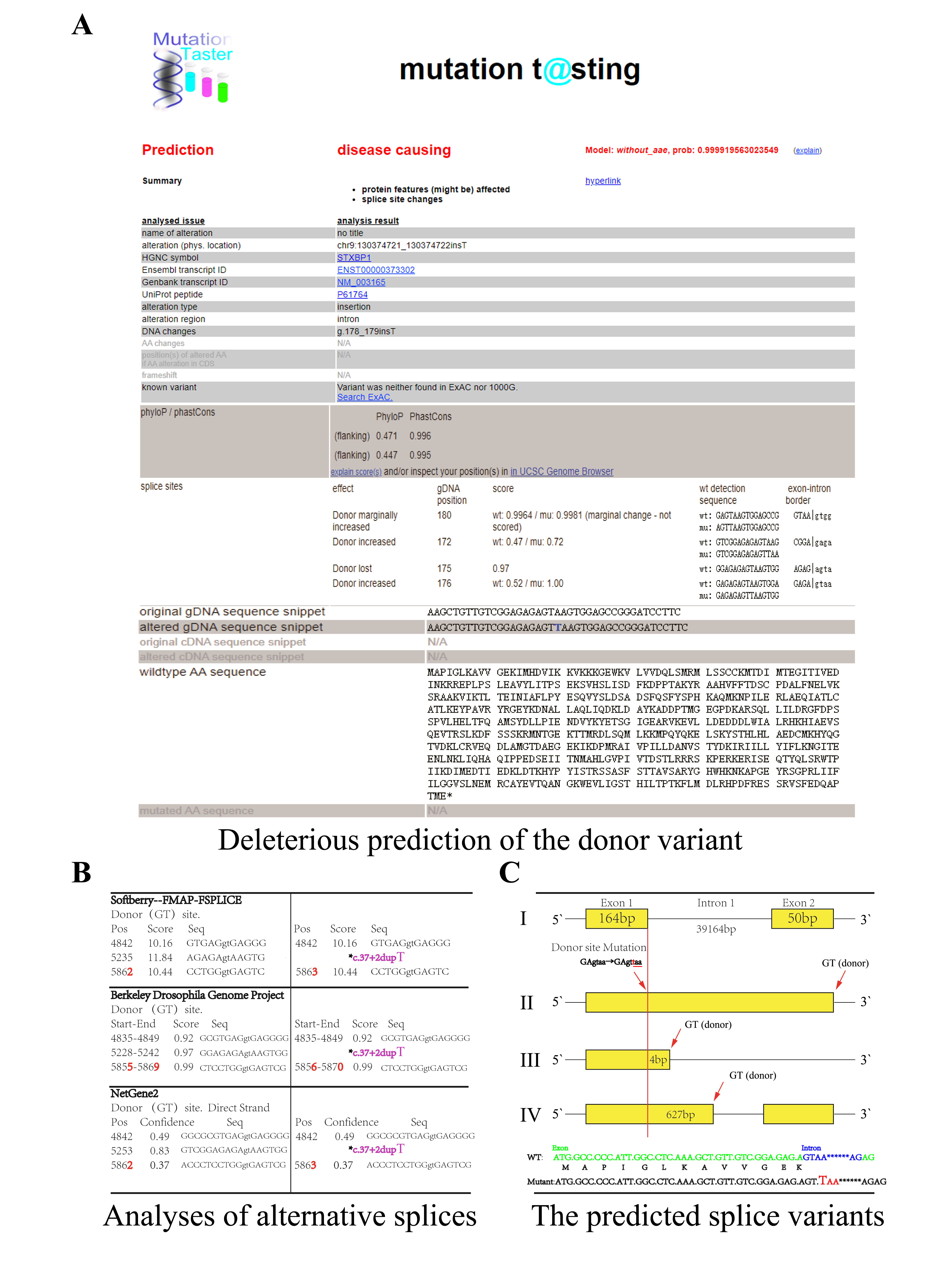

Supplement: Supplementary file 1 [file ijms-25-10983-s001.zip › Figure S3.jpg]
